# pH-Sensitive TRPC5 Is Differentially Expressed in Various Common Skin Tumors

**DOI:** 10.3390/biology14070823

**Published:** 2025-07-07

**Authors:** Lara Hopmann, Judith Heider, Dennis Niebel, Katja Evert, Florian Zeman, Christoph M. Hammers, Tobias Ettl, Christoph Brochhausen, Stephan Schreml

**Affiliations:** 1 Department of Dermatology, University Medical Center Regensburg, Franz-Josef-Strauß-Allee 11, 93053 Regensburg, Germany; lara.hopmann@stud.uni-regensburg.de (L.H.); judith.heider@ukr.de (J.H.); dennis.niebel@ukr.de (D.N.); christoph.hammers@ukr.de (C.M.H.); 2 Institute of Pathology, University of Regensburg, Franz-Josef-Strauß-Allee 11, 93053 Regensburg, Germany; katja.evert@ukr.de; 3 Center for Clinical Studies, University Medical Center Regensburg, Franz-Josef-Strauß-Allee 11, 93053 Regensburg, Germany; florian.zeman@ukr.de; 4 Department of Maxillofacial Surgery, University Medical Center Regensburg, Franz-Josef-Strauß-Allee 11, 93053 Regensburg, Germany; tobias.ettl@ukr.de; 5 Institute of Pathology, Medical Faculty Mannheim, University Heidelberg, Theodor-Kutzer-Ufer 1-3, 68167 Mannheim, Germany; christoph.brochhausen-delius@umm.de

**Keywords:** TRPC5, skin tumors, melanoma, squamous cell carcinoma, basal cell carcinoma

## Abstract

Transient receptor potential classical or cation channels (TRPCs) are integral to tumor biology, particularly in maintaining Ca^2+^ homeostasis within cancer cells. TRPC5, a pH-sensitive member of this family, may act as a signaling molecule in the altered microenvironment of solid tumors, which are characterized by an inverted pH-gradient—with decreased extracellular and increased intracellular pH—that promotes tumor progression. This study addresses a gap in the field, as there is currently limited research on TRPC5, particularly regarding its potential role as a tumor marker. While TRPCs are known to be involved in cancer biology, the specific role of TRPC5 in solid tumors, including its potential role as a diagnostic marker, remains largely unexplored. This study is the first to examine TRPC5 expression profiles in common skin cancers, including basal cell carcinoma (BCC), squamous cell carcinoma (SCC), malignant melanoma (MM), and nevus cell nevi (NCN). Our findings reveal that the frequency of TRPC5 expression in BCC is significantly lower compared to SCC and epidermal portions of NCN and MM. These results suggest that TRPC5 could serve as an immunohistochemical marker to distinguish SCC from BCC. Additionally, this study lays the groundwork for future research into the role of TRPC5 in tumor progression and metastasis, especially since BCCs, which rarely metastasize, are predominantly negative for TRPC5.

## Simple Summary

Transient receptor potential classical or cation channels (TRPCs) are found in cell membranes, one of their functions being the regulation of Ca^2+^ homeostasis in cells. TRPC5 is a pH-sensitive protein and acts as a signaling molecule in the altered microenvironment of solid tumors, which show an inverted pH-gradient—with decreased extracellular and increased intracellular pH. This study addresses the lack of knowledge on TRPC5 in skin, especially its potential role in tumors. Here, we examine for the first time the TRPC5 expression profiles in skin tumors, i.e., basal cell carcinoma (BCC), squamous cell carcinoma (SCC), malignant melanoma (MM), and nevus cell nevi (NCN). We show that the frequency of TRPC5 expression in BCC is significantly lower compared to SCC and portions of NCN/MM. This suggests that TRPC5 could be an immunohistochemistry marker to distinguish SCC from BCC and could partially explain the low likelihood of spread in BCC as compared to SCC/MM along with other data from our previous reports on pH-sensitive receptors in these entities. Hence, this study gives rise to future research on the role of TRPC5 in tumor progression and metastasis, especially since BCCs, which rarely metastasize, are predominantly negative for TRPC5 and multiple other pH-sensitive proteins.

## 1. Introduction

In recent decades, the incidence of skin cancer has increased [1]. Skin diseases like malignant melanoma (MM) and non-melanoma skin cancer (NMSC) are highly prevalent among the general population. Approximately 99,780 new cases of MM are projected to be diagnosed in the United States in 2022 [2]. The combined incidence of in situ and invasive melanoma experienced a substantial rise over the last decades. This can be primarily attributed to lifestyle changes and increased travel [3,4]. However, to some extent these numbers are also due to more frequent screening and early diagnosis of lesions [5]. MM poses a significant risk of metastasis, accounting for approximately 90% of skin cancer-related deaths. However, recently developed strategies to activate innate immunity (antibodies directed against PD1 and CTLA4) have drastically improved prognosis. The group of NMSCs primarily consists of basal cell carcinomas (BCCs) and squamous cell carcinomas (SCCs) [1,6]. In contrast to BCCs, however, advanced SCCs sometimes metastasize, whereas this is an absolute rarity in BCCs. Nevus cell nevi (NCNs) are benign melanocytic lesions that typically do not necessitate any intervention, but malignant melanoma can arise from dysplastic NCNs or develop de novo [7,8].

In a physiological microenvironment, the intracellular pH (pHi), ranging from 6.9 to 7.2, is lower than the extracellular pH (pHe), ranging from 7.2 to 7.4 [9]. Tumors are known to alter the physiological microenvironment. Standard stromal cells maintain their pHi within the narrow range of 6.9 to 7.2, but solid tumors display reduced pHe while maintaining an elevated alkaline pHi (7.2–7.4) [10,11]. This disruption in pH balance, referred to as a reserve pH gradient (pHe < pHi) or pH dysregulation, has been identified as a characteristic trait of cancer [9]. The tumor microenvironment accumulates acidic metabolic waste products (e.g., lactate, Waarburg effect) due to insufficient blood perfusion, leading to hypoxia, as well as inflammation and heightened metabolic activity [12,13,14].

The control of pH levels within both the pHi and pHe of tumors is heavily reliant on other transporters like carbonic anhydrases, the sodium/hydrogen antiporter NHE1, V-ATPases, and numerous others [14]. For normal healthy cells, this pH condition is harmful and usually leads to apoptosis, while tumors manage to not only prevent apoptosis triggered by acidosis but also manage to proliferate, adapt their metabolism, migrate, and invade. Furthermore, they exploit this condition to inhibit the natural immune response [15,16,17]. This manipulation of the pH gradient contributes to increased tumor growth and metastasis [18].

Consequently, the change in pH also affects various molecules and activates receptors sensitive to protons. These pH-sensitive receptors and channels include TRPCs, certain G protein-coupled receptors (GPCRs), transient receptor potential vanilloid channels (TRPVs), TWIK-related acid-sensitive potassium channels (TASKs), and acid-sensitive ion channels (ASICs) [19,20,21,22,23]. As TRPCs are involved in the regulation of calcium interchange, dysregulated TRPC5 activation may lead to altered calcium levels in cancer cells. The change in calcium concentration could trigger signal pathways that are closely linked to the advancement of melanoma, particularly the development of resistance to chemotherapy. It also effects the tumor microenvironment and the vascular network, as well as wound healing and angiogenesis [24,25,26].

TRPC5 is a pH-sensitive cation channel from the family of TRPCs (transient receptor potential classical or cation channels). TRPCs are located in the plasma membrane, and they are activated through various stimuli and a mechanism dependent on phospholipase C and G-protein-coupled receptors, which transduces the signal [27]. The activation of TRPC5 leads to calcium influx, thereby increasing the intracellular calcium concentration [28]. They play a role in tumor metabolism, especially in the Ca^2+^ homeostasis of tumor cells, which could affect tumor metabolism [19,27,29]. As TRPC5 is sensitive to local pH changes, it is particularly relevant in the context of the tumor microenvironment, and maybe also in tumor growth and spread.

Despite the fact that the information known about the role of TRPC5 in skin tumors is slim to none, studies show that TRPC5 plays a role in the pathological development of keratinocytes, e.g., in psoriasis [30]. It is also known that healthy and neoplastic keratinocytes differ in their levels of internal calcium concentration and their reaction to changing calcium levels [31]. TRPC5 expression is also related to chemotherapy-resistant breast cancer cells and might be a marker for chemoresistance in breast cancer cells and colorectal cancer cells [32,33,34]. Furthermore, the overexpression of TRPC5 leads to the transcription of p-glycoprotein, which pumps numerous substances, including chemotherapeutics, out of the cells [35]. Thus, drugs targeting the regulation of TRPC5 may be relevant for treating drug resistance. This could be important in patients with metastatic melanoma [36], but also SCC and, in rare cases, BCC. New studies show that TRPC5 is also sensitive to oxidative stress in the tumor microenvironment [37].

## 2. Materials and Methods

For our staining, paraffin-embedded tissue samples were used from the dermatopathological routine of the Department of Dermatology, University Medical Center Regensburg. All the samples were aged over 10 years, rendering them permissible for use under German legal regulations. The evaluated samples were procured from regions affected by localized skin tumors in patients.

### 2.1. Immunohistochemistry

Paraffin-embedded tissue samples were cut into 3 μm sections with a microtome and then affixed to microscope slides. Additionally, every slide underwent staining with hematoxylin and eosin. All staining procedures were carried out at room temperature. To guarantee comparability and reduce variations in staining, the antibody was incubated overnight at 4 °C to bind better.

After deparaffinization, tissue sections were incubated at 75 °C for 30 min to ensure proper sealing. After this, we rehydrated the tissue with decreasing alcohol concentrations according to our guideline:

This included 3 × xylol for 10 min, 2 × 100% ethanol for 10 min, 2 × 96% ethanol for 10 min, and 2 × 70% ethanol for 10 min. The tissue sections were placed in 3% $H_{2}O_{2}$ for 10 min to block endogenous peroxidase activity. To produce 3% $H_{2}O_{2}$, 3 mL of 30% $H_{2}O_{2}$ was mixed (Roth, Karlsruhe, Germany, No. 8070.1) with 100 mL of 70% ethanol. At the same time, a pH 6 citrate buffer (Zytomed, Berlin, Germany, ZUC028-500) was boiled for 30 min.

The slides underwent a wash with distilled water and were subsequently subjected through a 20 min boiling process in the precooked citrate buffer, followed by cooling on ice for a 20 min period. Following that, they were moved to the phosphate buffer solution (PBS) (Sigma-Aldrich, Darmstadt, Germany, No. D8537) and left there for a duration of 10 min. Subsequently, protein-blocking solutions (Zytomed, ZUC007-100) were put onto the slides for 5 min, before they were moved to the washing buffer solution (Zytomed, ZUC020-500) for another 5 min. The tissue samples were incubated overnight at 4 °C with the primary rabbit anti-human TRPC5 polyclonal antibody (Alomone labs, Israel, Jerusalem, 0.85 mg/1 mL Anti-TRPC5 antibody RRID: AB_2040241).

The antibody was diluted with antibody diluent (Zytomed, REF ZUC025-500) 1:400 before it was added to the slides. The next day, the slides were washed three times with a washing buffer solution, before the tissue sections were incubated with a secondary antibody (Nichirei, Histofine simple stain rabbit, Tokyo, Japan, No. 414141F) for 30 min. Afterwards, they underwent another three washing sessions with the washing buffer solution. To make the staining action visible, the next step was to apply a chromogen solution (AEC) to the tissue sections.

The AEC substrate must be freshly prepared for each staining process, as the substrate is unstable. Therefore, we added 20 μL of an AEC chromogen to 10 mL of an AEC substrate buffer and mixed it well (Zytomed, No. ZUC42-500). As soon as the desired intensity of coloration was achieved, the reaction was stopped with distilled water. The counterstaining was performed with Mayer’s Haemalaun for 1–2 min (Roth, No. T865.3) and was blued with tap water. The PreciPoint M8 system was used to scan the slides, and image editing was performed using ViewPoint Online (PreciPoint, M8 system, Garching bei München, Germany).

### 2.2. Western Blot

To prove that the TRPC5 antibody binds specifically, an additional Western blot analysis was performed on a protein basis. The proteins were denatured through the addition of DTT with boiling at 70 °C. To separate the proteins, we used a 10% mini-PROTEAN TGX stain-free gel (BioRad, Hercules, CA, USA, No. 4568126) and added 10 μL of protein per lane. After blotting the proteins onto PVDF membranes (BioRad, No. 1620261), the membranes were blocked with 10% goat serum in a PBTS buffer for 2 h. The primary antibody (1:750 in 5% goat serum in PBST) was then incubated overnight at 4 °C. The next day, the secondary antibody was added to the blots for 1 h (BioRad, StarBright 700 goat anti-rabbit, 1:10,000 in PBST, No. 12004162). After four washes with PBST and PBS (Gibco, Thermo Fisher Scientific, Waltham, MA, USA, No. 14190-094), the blots were evaluated using a CemiDocGo (BioRad).

### 2.3. Immunfluorescence

Immunofluorescence staining for TRPC5 detection was performed using Alexa Fluor 594 and imaging was conducted with the Texas Red filter on a Keyence microscope. Goat anti-rabbit Alexa Fluor 594 (CellSignaling 8889S, Denver, MA, USA) was used as the secondary antibody at a dilution of 1:5000, and DAPI was applied for nuclear staining. For deparaffinization, tissue sections were treated in xylene (3 × 5 min), followed by 100% ethanol (2 × 5 min), 96% ethanol (2 × 5 min), and 70% ethanol (2 × 5 min). Subsequently, the sections were rinsed in PBS. To prepare the samples, slides were placed in a rack and immersed in a mixture of citrate buffer (pH 6) and Tris–EDTA buffer (pH 9), then heated in a steamer for 25 min. After cooling at room temperature for 25 min, blocking and incubation with primary antibodies were performed.

Slides were washed once with PBS and incubated in a Tris–glycine buffer for 15 min to reduce autofluorescence, followed by another PBS wash. To block nonspecific binding, sections were incubated with 4% BSA in PBST for 30–60 min. Primary antibody incubation was carried out overnight at 4 °C in a humidified chamber with the antibodies diluted in 1% BSA in PBST.

On the following day, all steps were performed under light-protected conditions. Slides were washed 3 × 5 min in PBST (0.1% Tween-20) and incubated in 1% BSA in PBST for 15 min. The secondary fluorescent antibody, diluted in 1% BSA in PBST, was applied for 30 min at room temperature, followed by 3 × 5 min washes in PBST. Nuclei were stained with DAPI at a dilution of 1:50,000 (0.5 µL in 5000 µL PBS) for 1–3 min, and slides were washed again 3 × 5 min in PBST. Finally, sections were covered with a special fluorescence-covering medium. This protocol ensured optimal fluorescence signal detection and preservation for imaging. This experiment was conducted to confirm the specific binding of TRPC5.

### 2.4. Scoring

Experienced dermatopathologists from the Department of Dermatology and the Institute of Pathology at the University Medical Center Regensburg conducted visual assessments of the staining. They used the epidermis as a reference to evaluate staining intensity. Sections were categorized as follows: ++ for strong positive reactions (positivity or high-intensity staining was observed in over 80% of cells), + for weakly positive or partially positive reactions (20–80% of cells displaying weak or partially strong staining), and—for negative reactions (weak staining was observed in less than 20% of cells). Samples that showed inconsistent staining across tumor samples and reduced expression levels in deeper tissue layers were classified as weakly positive.

### 2.5. Statistics

Initially, ratings for all entities were compared using Kruskal–Wallis tests. Pairwise comparisons were performed using Bonferroni tests. For NCNs and MM, separate analyses were conducted on epidermal and dermal portions.

Subsequently, pairwise comparisons were made between NCN dermal and epidermal parts with MM dermal and epidermal parts, as well as between BCC and MM epidermal and dermal parts and BCC and NCN dermal and epidermal parts. SCC was compared to MM dermal and epidermal layers and to NCN dermal and epidermal layers. Basal cell carcinomas (BCCs) and squamous cell carcinomas (SCCs) were also compared. NCN values for each protein were determined using a Mann–Whitney U test. The results are presented as exact significance (shown as 2*(1-tailed significance), not corrected for ties, for BCCs vs. SCCs and epidermal portions of NCN/MMs) or asymptotic significance (2-tailed, for dermal portions of NCN/MMs).

## 3. Results

### 3.1. Controls

**Figure 1:**
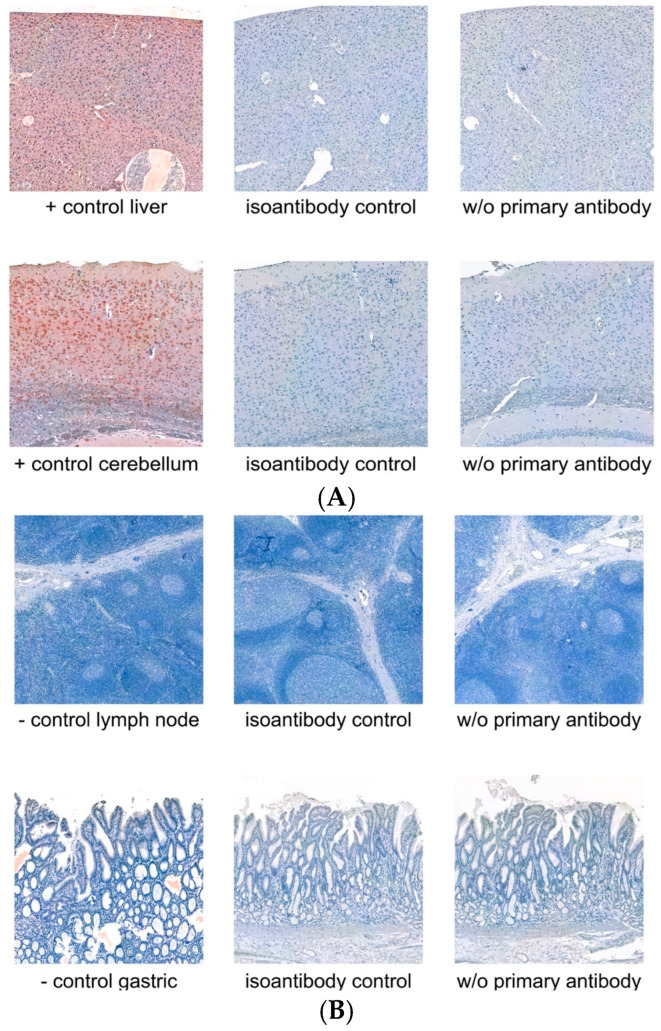
(**A**,**B**) Tissue control for the immunohistochemical staining of TRPC5. (**A**) Positive controls for TRPC5 consisted of cerebellum and liver tissues, which demonstrated specific staining. In relation to this, staining with isoantibody control and without primary antibody is shown. (**B**) Negative controls for TRPC5. Lymph node and gastric tissues were used as negative controls. No reaction is revealed in both positive controls and isoantibody controls.

Immunohistochemical staining was validated on skin samples. Negative controls were conducted without primary antibodies and included the use of isoantibodies (detailed in the methods below). For positive controls, the liver and cerebellum were stained (Figure 1A), while for negative controls, we used lymph node and gastric tissues (Figure 1B).

### 3.2. Immunfluorescence

**Figure 2:**
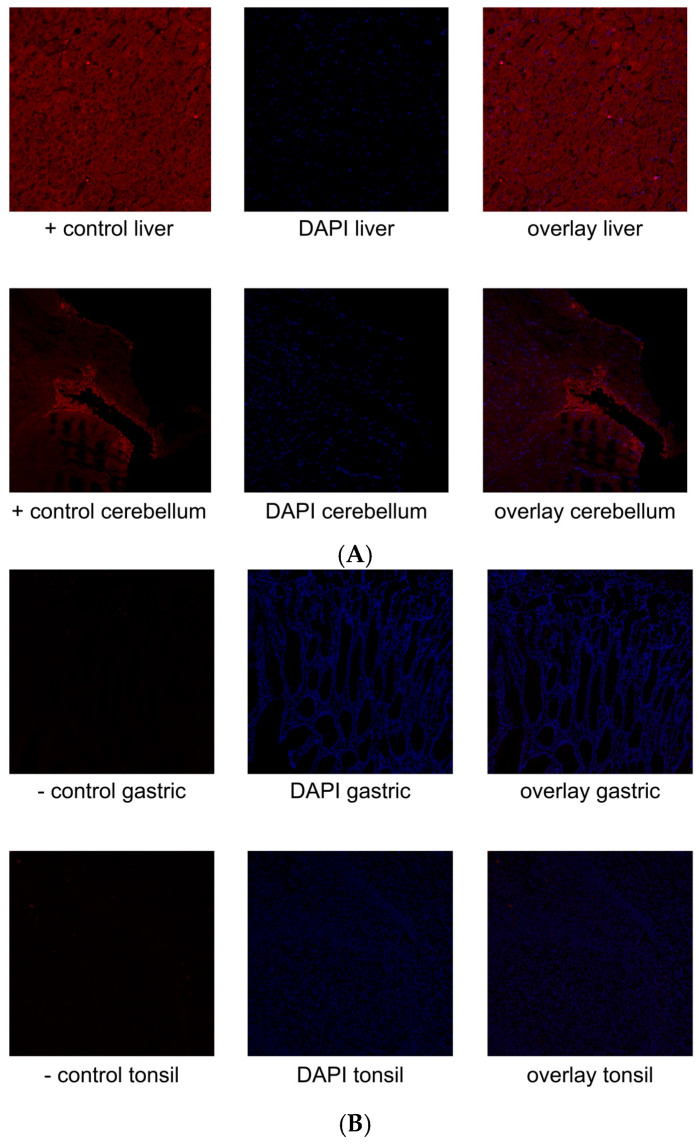
(**A**,**B**) Tissue control for specific binding of TRPC5 with immunofluorescence. (**A**) Positive controls for TRPC5. Liver and cerebellum tissues functioned as positive controls for TRPC5. (**B**) Negative controls for TRPC5. Gastric and tonsil tissues were used for negative controls. No reaction is shown.

Using immunofluorescence staining, we successfully demonstrated the specific binding of the TRPC5 antibody. Clear fluorescence signals were observed in the samples, confirming the presence and localization of TRPC5. Figure 2A shows positive controls using the liver and cerebellum, while for negative controls, demonstrated in Figure 2B, gastric and tonsil tissues were stained. For each control, we present an image in Figure 2A,B showing secondary antibody staining, a separate image displaying nuclear staining with DAPI, and a merged overlay image.

### 3.3. Western Blot

**Figure 3:**
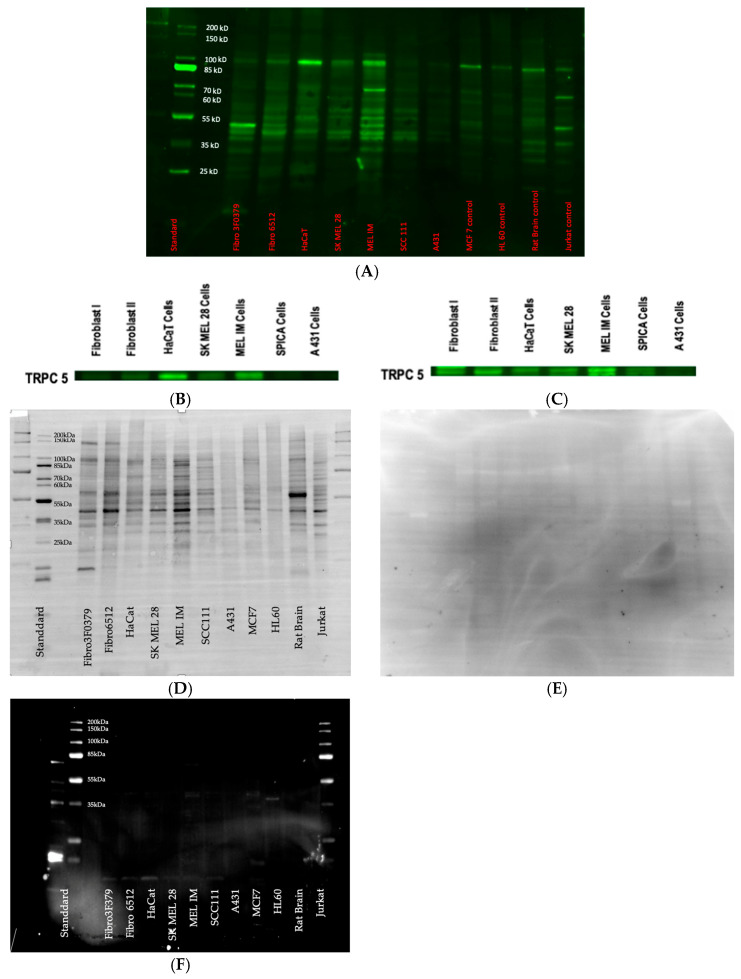
(**A**–**F**) The results of the Western blot are presented in an uncropped and a cropped version, as well as a stain-free blot. (**A**) Uncropped Western blot of TRPC5 antibody. Determination of the specificity of TRPC5 by Western blot at a concentration of 10 μg per lane in fibroblasts, HaCat cells, SK MEL 28 cells, MEL IM cells, SPICA cells, and A431 cells compared to the positive controls MCF 7, HL 60, Rat Brain, and Jurkat. Gel: 10% SDS (TGX Stain-Free Precast Gel); primary antibody: Anti-TRPC5 1:500, about 100 kDa; secondary AB: anti-rabbit 1:10,000 (StarBright 700, BioRad); marker: Bio Lab. (**B**) Cropped Western blot at 100 kDa. (**C**) Cropped Western Blot at 50 kDa. (**D**) Western Blot stain-free Blot after separation of the proteins and blotting. (**E**) This membrane shows that protein binding is completely blocked by incubation with the IgG isotype control and therefore no unspecific binding of the TRPC 5 antibody takes place. (**F**) Western Blot with control peptide. The antibody does not bind unsepcifically.

The presence of the primary antibody TRPC5 was confirmed via Western blot (Figure 3A–E). The results indicate the successful detection of TRPC5 and suggest that TRPC5 binds specifically under the conditions tested, supporting its specificity in this assay. A band was observed at approximately 50 kDa, accompanied by another band near 100 kDa. According to the manufacturer’s data sheet, the expected molecular weight of TRPC5 is around 100 kDa. The TRPC subfamily consists of seven members (TRPC1-7). Similarly to other members of the TRP family, they contain six transmembrane domains with an ion channel located between the fifth and sixth domain. The N- and C-termini of TRPCs harbor various functional domains, including ankyrin repeats, calmodulin-binding sites, phosphorylation sites, and interaction sites for other molecules such as Homer, Orai, STIM1, Junctate, and the IP3 receptor (IP3R). This complexity, particularly the phosphorylation and the presence of N- and C-termini in TRPCs, complicates the precise identification of bands at 100 kDa. A datasheet for the TRPC5 antibody displays a pattern in the Western Blot that closely resembles the one observed in our experiments.

### 3.4. Samples

**Figure 4:**
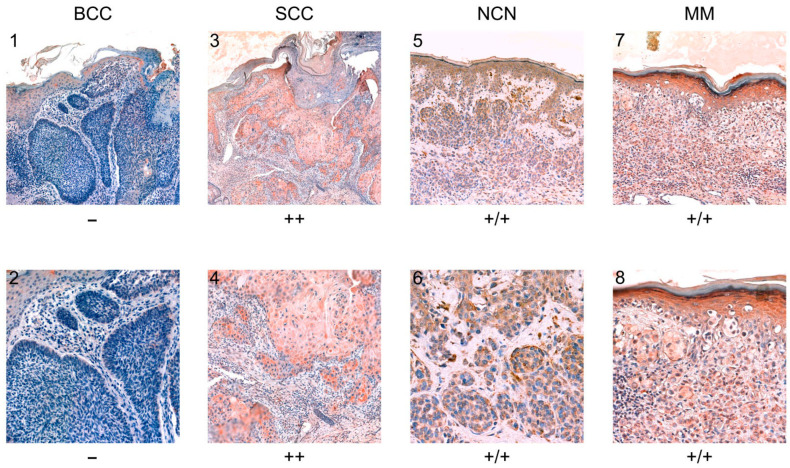
(**1**–**8**) Representative immunohistology staining for TRPC5 in BCC, SCC, NCN, and MM. The cells in BCC show a negative reaction, which means less than 20% of the tumor cells are evaluated as positive (examples are presented in picture (**1**,**2**), patient number 17, slide number BCC_2039_09, see Table S1). The tumor cells in SCC show strong positive staining (examples are shown in picture (**3**,**4**), patient number 11, slide number SCC_2908_09, see Table S2). The dermal and epidermal sections of NCN express a positive reaction (examples are presented in picture (**5**,**6**), patient number 11, slide number NCN_40_08, see Table S3). The dermal and epidermal parts of MM show positive staining in both parts as well (examples are shown in picture (**7**,**8**), patient number 9, slide number MM_253_12, see Table S4). All stains of BCC, SCC, NCN, and MM are shown in Supplementary Figures S1–S9.

To evaluate TRPC5 expression levels, we primarily gathered 30 samples of BCC, 33 samples of SCC, 19 samples of NCN, and 27 samples of MM. However, because of processing issues such as overstaining or very faint staining, a reduced number of samples fulfilled the evaluation criteria as detailed in the Methods Section. Furthermore, some samples lacked tumor formations or tumor cell nests, further reducing the number of evaluable samples as outlined: BCC n = 27, SCC n = 26, NCN n = 14, and MM n = 27. Figure 4 displays a panel of representative staining. Detailed staining and scoring results can be found in Supplementary Figures S1–S9 and Tables S1–S5, and clinical data including information about sex and age can be found the Supplementary Material, Table S6.

### 3.5. BCC

We analyzed twenty-five nodular BCCs, one superficial BCC and one mixed form (partially nodular and sclerosing). In 48.1% of the samples, a negative reaction for TRPC5 was observed. In all, 51.9% of the tissue samples showed a weak positive reaction, with 20–80% of cells displaying weak or partially strong staining, and zero samples were classified as strong positive, with >80% of cells being positive (Figure 4; Supplementary Figures S1 and S2; Tables S1 and S5).

### 3.6. SCC

Most of the 26 squamous cell carcinoma (SCC) samples demonstrated positive staining, with 53.8% being strong positive and 38.5% being weak positive. The two remaining specimens (7.7%) revealed a negative reaction (Figure 4; Supplementary Figure S3 and S4; Tables S2 and S5).

### 3.7. NCN

The samples were evaluated by dividing them into epidermal and dermal sections. Weak positive staining in both the epidermal and dermal section was observed in three out of fourteen NCNs. Three specimens appeared to be strongly positive in the dermal and epidermal part, and one sample was negative in both sections. In the epidermal sections, five out of fourteen (35.7%) samples exhibited strong positive staining, eight out of fourteen (57.1%) showed weak positive staining, and only one sample (7.1%) was rated as negative. In the dermal sections, two out of fourteen samples (14.3%) demonstrated a strong positive reaction, seven out of fourteen (50.0%) showed weak positive staining, and five out of fourteen (35.7%) displayed a negative reaction (Figure 4; Supplementary Figure S5; Tables S3 and S5).

### 3.8. MM

For MM, 37.1% of the samples showed weak positive staining in both the dermal and epidermal part, and 12.5% had a strong positive reaction in the epidermal and dermal part. A total of 29.6% showed a negative reaction in the epidermal and dermal layer.

**Figure 5:**
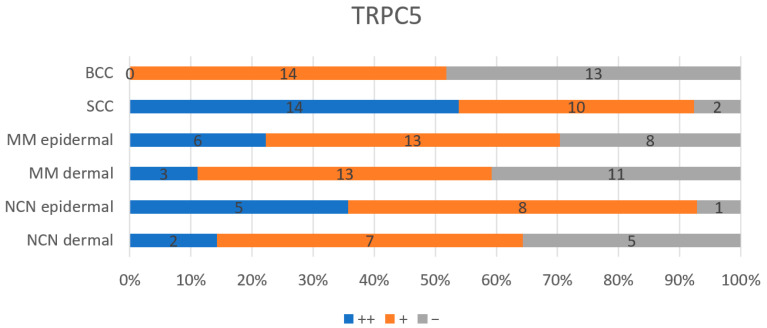
Summary of immunohistochemical scoring results for TRPC5 on BCCs, SCCs, NCN, and MMs. ++/blue bar: strongly positive staining, with >80% of cells being positive and/or high staining intensity; +/orange bar: 20–80% of cells are weakly positive; gray bar: negative reaction, which means <20% of cells had weak staining. MM and NCN are subdivided into dermal and epidermal parts. Numbers in bars represent the occurrence of the particular score. For detailed information on the individual scores, see Supplementary Tables S1–S5.

Three of the twenty-seven specimens were weakly positive in the dermal layers and strongly positive in the epidermal layers, and two out of twenty-seven were negative in the dermal parts and weakly positive in the epidermal parts (Figure 5, Supplementary Figure S6 and S7, and Tables S4 and S5).

### 3.9. Statistical Analysis of TRPC5—Comparison of All Entities

TRPC5 expression was significantly lower in basal cell carcinomas compared to squamous cell carcinomas (*p* < 0.001 Kruskal–Wallis and post hoc Bonferroni tests), as well as between BCC and MM epidermal section (*p*-value 0.039 Kruskal–Wallis and post hoc Bonferroni tests) and between BCC and NCN epidermal section (*p*-value < 0.001 Kruskal–Wallis and post hoc Bonferroni tests). A significant lower signal of TRPC5 was also found in dermal and epidermal parts of MM in comparison to SCC (*p*-value < 0.001 and <0.007 Kruskal–Wallis and post hoc Bonferroni tests) and the dermal section in NCN compared to SCC (*p*-value < 0.005 Kruskal–Wallis and post hoc Bonferroni tests). No significant differences in expression of TRPC5 were found between BCC and MM dermal section, BCC and NCN dermal section, MM dermal/epidermal and NCN dermal/epidermal part, as well as NCN epidermal part compared to SCC (Table 1 and Table S5).

## 4. Discussion

A key result of our study was that TRPC5 expression was less frequent in basal cell carcinoma (BCC) than in squamous cell carcinoma (SCC). BCCs completely lacked pH sensitive TRPC5 expression in nearly half of the cases examined in this study. Additionally, expression of TRPC5 in BCC was significantly lower in comparison to epidermal portions of MM and NCN. This differential expression pattern represents a significant immunohistochemical characteristic.

Mutation frequencies of TRPC5 in the studied tumor entities were analyzed using cBioPortal data. An about 13% mutation frequency was found for TRPC5 in MM (Supplementary Figure S8). For SCC, two studies showed a slightly lower mutation frequency in comparison to BCC (Supplementary Figure S9). Our study found that the lower protein expression detected in BCCs relative to SCCs may reflect underlying post-transcriptional regulatory differences.

Our results are comparable to the outcomes of observations made for TRPC4, GPR31, GPR151, TASK1, TASK3, and ASIC2 in previous research [9,19]. Despite the limited data on TRPC5 expression in cutaneous tumors, evidence from other tissues indicates its involvement in cancer progression, supporting its potential role as a therapeutic target. [38]. As said, pH activates a plethora of GPCRs and TRPCs, so these proteins might be of clinical relevance. For instance, englerin A is a notable example worth examining: At effective concentrations, it can activate ion channels of TRPC4 and TRPC5. Furthermore, it limits the proliferation of tumor cell lines with an increased TRPC4/TRPC5 presence. Despite its potency, in rodent serum, englerin A exhibits a chemical instability and pronounced toxicity toward rodents [39,40]. Tonantzitlolone (TZL) has shown at least superficially comparable effects to englerin A [41]. Nevertheless, TZL is chemically distinct, potentially offering innovative approaches due to the recognized severe in vivo toxicity of englerin A in healthy rodents, which poses a considerable challenge to its clinical development. Monoclonal antibodies directed against TRPC5 could serve as a less toxic and more specific option to treat irresectable or rare metastasized cases. Using TRPC5 as an immunohistochemical marker, however, can potentially also serve as an option in hard-to-distinguish entities of adnexal tumors. Further studies on these rare tumors are needed to establish this marker.

Moreover, TRPC5 is involved in the pathological development of keratinocytes and is inhibited in cells affected by psoriasis [30]. In epidermal cells, an elevation in intracellular calcium levels is a crucial event triggering their differentiation [31]. Calcium influxes facilitated by TRPC5 may play a significant role in the pathophysiology of epidermal keratinocytes and other skin diseases. It is also known that TRPC5 is associated with breast cancer cells and colorectal cancer cells [32,33]. Thus, drugs targeting the regulation of TRPC5 may be important for therapy against chemoresistance, which could also be relevant for skin cancer, especially irresectable or metastasized cases.

Considering these factors along with our findings, TRPC5 could be a potential diagnostic tool and drug target in skin cancer. However, further research is needed to clarify its role in the progression of skin tumors.

## 5. Conclusions

This study addresses the significant gap in research on TRPC5, despite its potential role as a signaling molecule in the altered microenvironment of solid tumors. Our findings revealed a notably lower expression of TRPC5 in basal cell carcinoma (BCC) in comparison to squamous cell carcinoma (SCC), with nearly half of the BCC cases showing no expression at all. Additionally, TRPC5 expression in BCC was significantly lower compared to the epidermal portions of malignant melanoma (MM) and nodular compound nevi (NCNs). These differential expression patterns highlight TRPC5 as a potential histological marker for distinguishing BCC from SCC and underscore the need for further studies to elucidate its functional role in tumor progression.

Firstly, our findings need to be validated with a larger sample size and by studying various patients, particularly focusing on BCC growth patterns, to better understand the distinct roles of TRPC5. Future studies might benefit from the automated evaluation of immunohistochemistry. Utilizing cultured cells with knockout and overexpression of TRPC5, subjected to different pHe conditions, could help elucidate this protein’s role in proliferation, migration, and cell survival. Following the identification of the expression profiles of selected proteins in a variety of cell models through qPCR and Western blot, a potential next step could involve knockdown (siRNA)/knockout (CRISPR/Cas9) and overexpression techniques combined with functional assays to examine these aspects at the cellular level, as previously suggested [20].

Since this is the first research to investigate the expression of TRPC5 in common skin tumors, our study is primarily descriptive. A particularly notable finding is the lower expression of TRPC5 in BCC compared to SCC. The expression in MM and NCNs were also lower. This outcome mirrors our earlier research on TRPC4, ASIC2, and GPR31, which also revealed a minimal signal in BCC and complete detection in SCC [9,23].

This makes the research on TRPC5 a further new diagnostic and histological instrument to differentiate between basal cell carcinoma and squamous cell carcinoma.

## Figures and Tables

**Table 1:** Testing results with Kruskal–Wallis test and post hoc Bonferroni comparison.

Pairs	*p*-Value	Adj. *p*-Value
BCC-MM dermal	0.351	1.000
BCC-NCN dermal	0.263	1.000
BCC-MM epidermal	0.039	0.585
BCC-NCN epidermal	0.001	0.018
BCC-SCC	<0.001	<0.001
MM dermal-NCN dermal	0.727	1.000
MM dermal-SCC	<0.001	0.002
NCN dermal-SCC	0.005	0.077
MM epidermal-NCN epidermal	0.125	1.000
MM epidermal-SCC	0.007	0.112
NCN epidermal-SCC	0.487	1.000

## Data Availability

Available upon reasonable request.

## Supplementary Materials

The following supporting information can be downloaded at: https://www.mdpi.com/article/10.3390/biology14070823/s1, Figures S1 and S2: immunohistochemistry for TRPC5 in BCC; Figures S3 and S4: Immunohistochemistry for TRPC5 in SCC, Figure S5: Immunohistochemistry for TRPC5 in NCN, Figures S6 and S7: Immunohistochemistry for TRPC5 in MM, Figure S8: TRPC5 mutation frequency in MM from cBioportal.org, Figure S9: TRPC5 Mutation frequency in non-melanoma skin cancer from cBiportal.org, Table S1: Scoring results for TRPC5 in BCC, Table S2: Scoring results for TRPC5 in SCC, Table S3: Scoring results for TRPC5 in NCN, Table S4: Scoring results for TRPC5 in MM, Table S5: Statistical analysis of TRPC5—comparison of all entities, Table S6: Patient Data, age and sex of the patient the tissue samples were taken from.
