# Supplementary material for: pH-Sensitive TRPC5 Is Differentially Expressed in Various Common Skin Tumors
Source: Biology (Basel). 2025 Jul 7;14(7):823. doi: 10.3390/biology14070823 (PMC12292088; doi:10.3390/biology14070823)
Supplement: Supplementary file 1 [file biology-14-00823-s001.zip › biology-3283759-supplementary.pdf]

Supplementary Figures S1-S2: Immunohistochemistry for TRPC5 in BCC. Scale bars represent 200 μm. In BCC, TRPC5 expression was weak positive for 14/27 samples (1,2,3,4,6,7,11,14,20,21,22,23,24,25,27) and 13/27 samples (5,8,9,10,12,13,15-19,23,26) showed a negative staining.

1=BCC_886_12, 2=BCC_5512_12, 3=BCC_8705_12, 4=BCC_9027_12, 5=BCC_12206_12, 6=BCC_12287_12, 7=BCC_14077_12, 8=BCC_223_09, 9=396_09, 10=BCC_1442_06, 11=BCC_1786_12, 12=BCC_5627_12, 13=BCC_845_09, 14=BCC_925_09, 15=BCC_1121_09, 16=BCC_1136_09, 17=BCC_2039_09, 18=BCC_2388_12, 19=BCC_2439_12, 20=BCC_252_06, 21=BCC_1408_12, 22=BCC_8880_12, 23=BCC_9013_12, 24=BCC_9463_12, 25=BCC_9672_12, 26=BCC_12207_12, 27=BCC_12273_12

Supplementary Figure S1: Immunohistochemistry for TRPC5 in BCC (part 1)

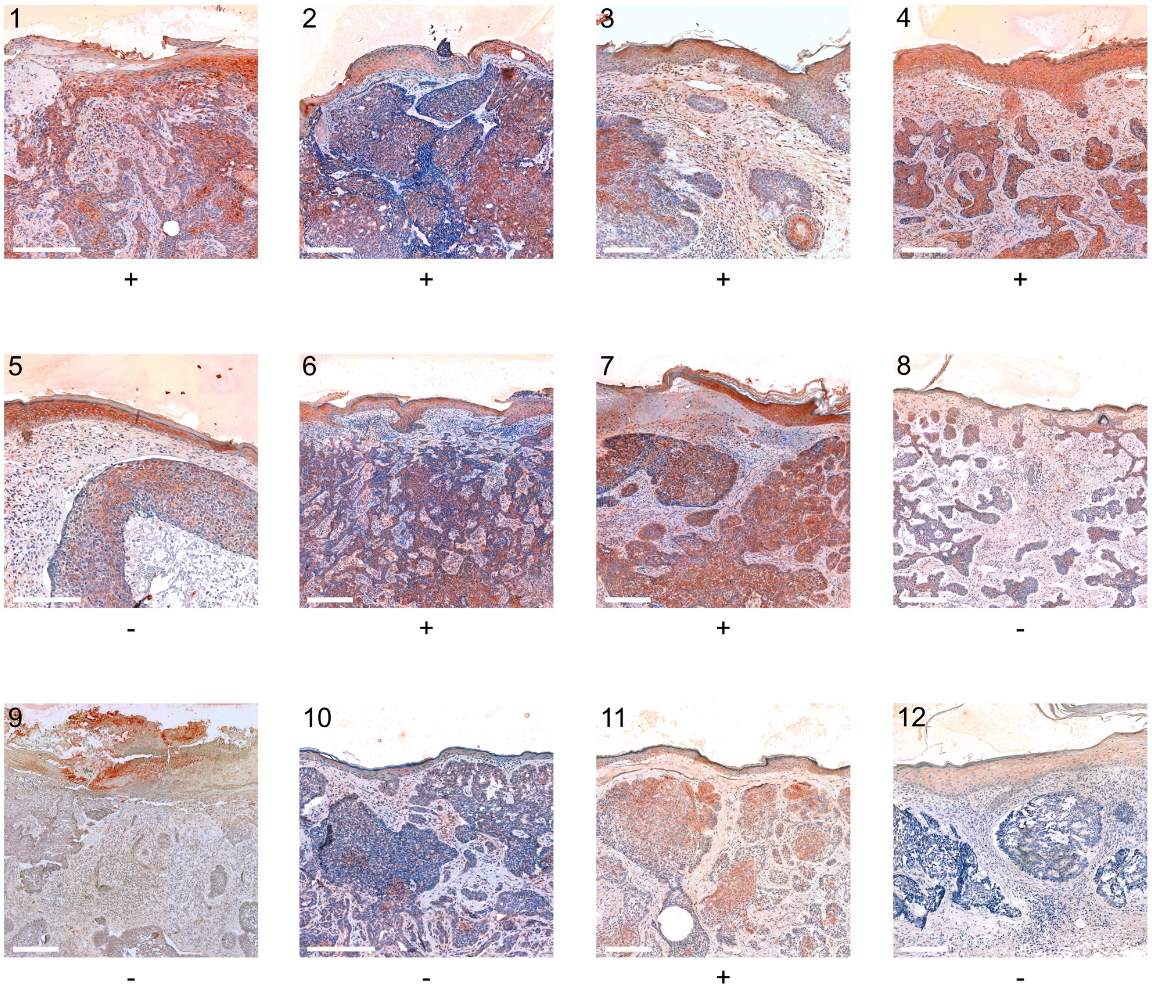

Supplementary Figure S2: Immunohistochemistry for TRPC5 in BCC (part 2)

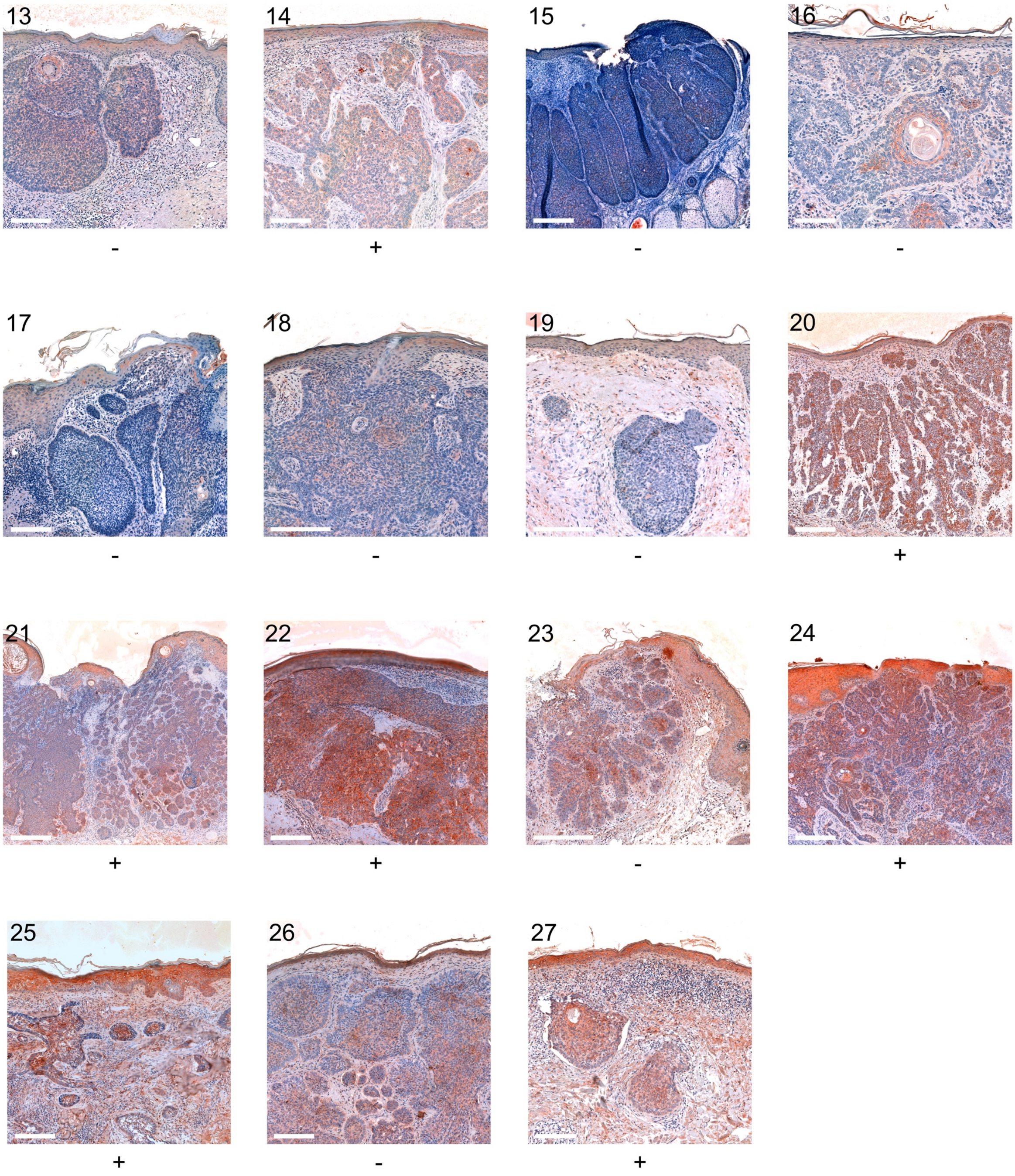

Supplementary Figures S3-S4: Immunohistochemistry for TRPC5 in SCC. Scale bars represent 200 μm.
SCC showed a mainly positive staining with 14/26 samples rated as strong positive (1,4,5,6,9,11,12,14,19,20,21,22,23,24) and 10/26 samples show a weak positive staining (3,7,8,10,13,15,16,17,25,26). Only two negative reactions are displayed (2,18).

1=SCC_363_12, 2=SCC_664_12, 3=SCC_902_12, 4=SCC_2090_12, 5=SCC_9314_09, 6=SCC_18330_12, 7=SCC_21605_12, 8=SCC_24081_12, 9=SCC_26097_12, 10=SCC_33497_12, 11=SCC_2908_09, 12=SCC_3385_06, 13=SCC_5682_08, 14=SCC_11284_06, 15=SCC_11486_06, 16=SCC_11824_06, 17=SCC_13207_06, 18=SCC_29707_12, 19=SCC_18011_06, 20=SCC_16467_06, 21=SCC_13529_06, 22=SCC_13160_12, 23=SCC_11190_06, 24=SCC_11160_09, 25=SCC_3385_06, 26=SCC_1984_12

Supplementary Figure S3: Immunohistochemistry for TRPC5 in SCC (part 1)

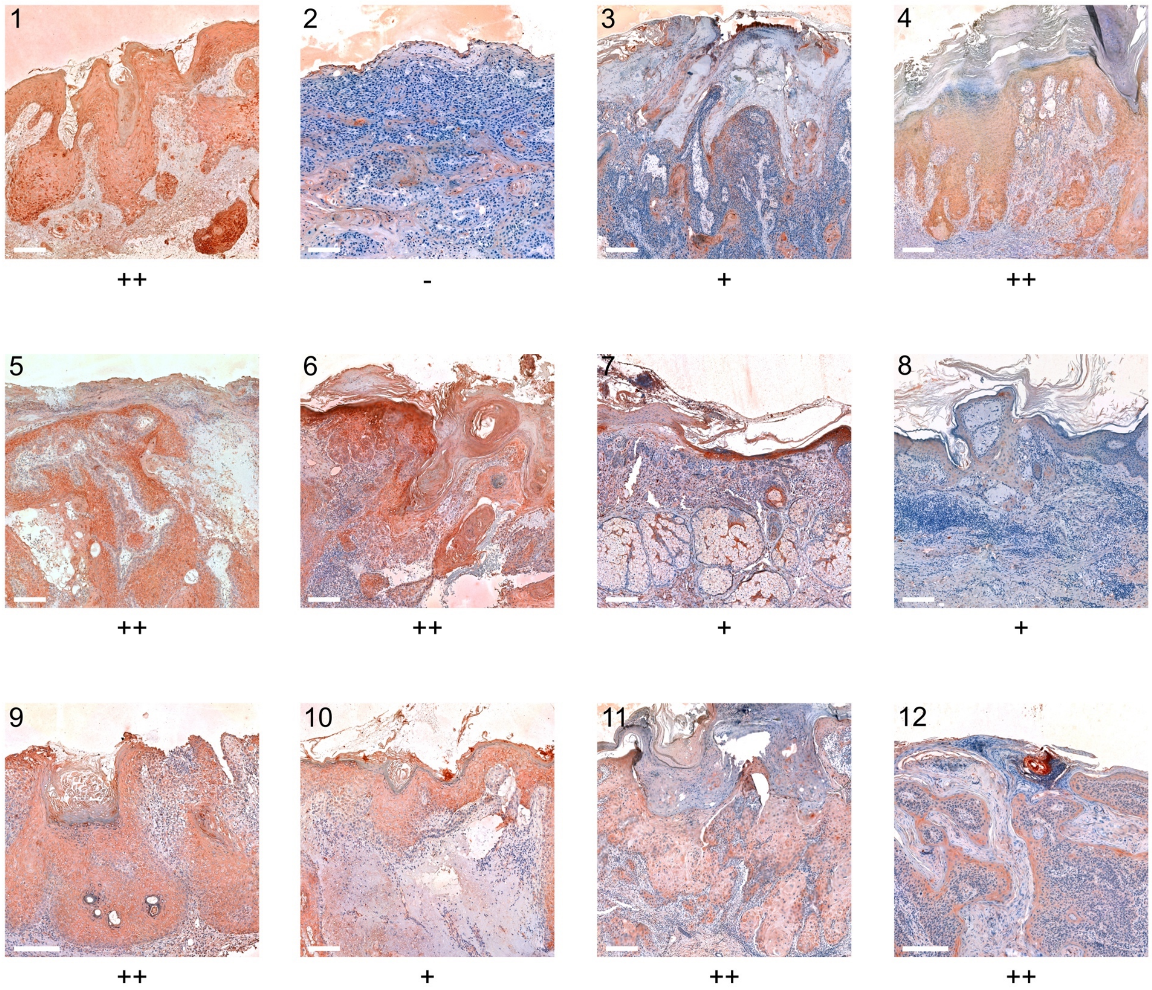

Supplementary Figure S4: Immunohistochemistry for TRPC5 in SCC (part 2)

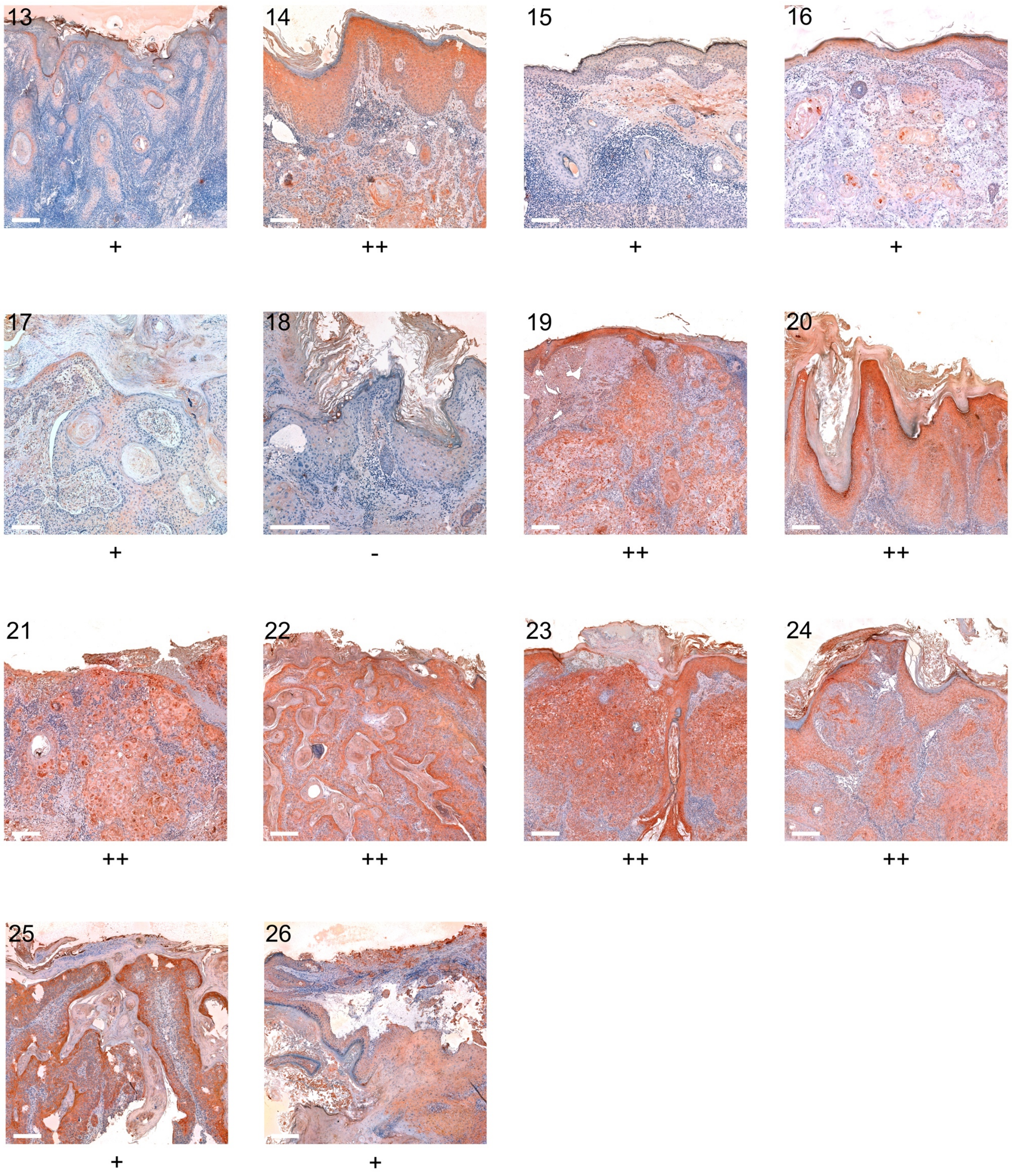

Supplementary Figure S5: Immunohistochemistry for TRPC5 in NCN. Scale bars represent 200 μm. In epidermal sections, 5/14 samples show a strong positive staining (1,2,7,8,14), 8/14 show a weak positive staining (3,5,6,9,10,11,12,13) and only one negative reaction is rated (4). The dermal parts expressed a strong positive reaction in two out of 14 samples (7,8) and 7/14 show a weak positive staining (1,2,6,9,11,13,14). 5/14 displayed a negative reaction.

1=NCN_412_12, 2=11394_12, 3=NCN_11491_12, 4=NCN_11602_12, 5=NCN_11704_12, 6=NCN_17735_09, 7=NCN_26174_12, 8=NCN_37_08, 9=NCN_38_08, 10=NCN_1471_06, 11=NCN_40_08, 12=NCN_290_11, 13=NCN_32_08, 14=NCN_192_12

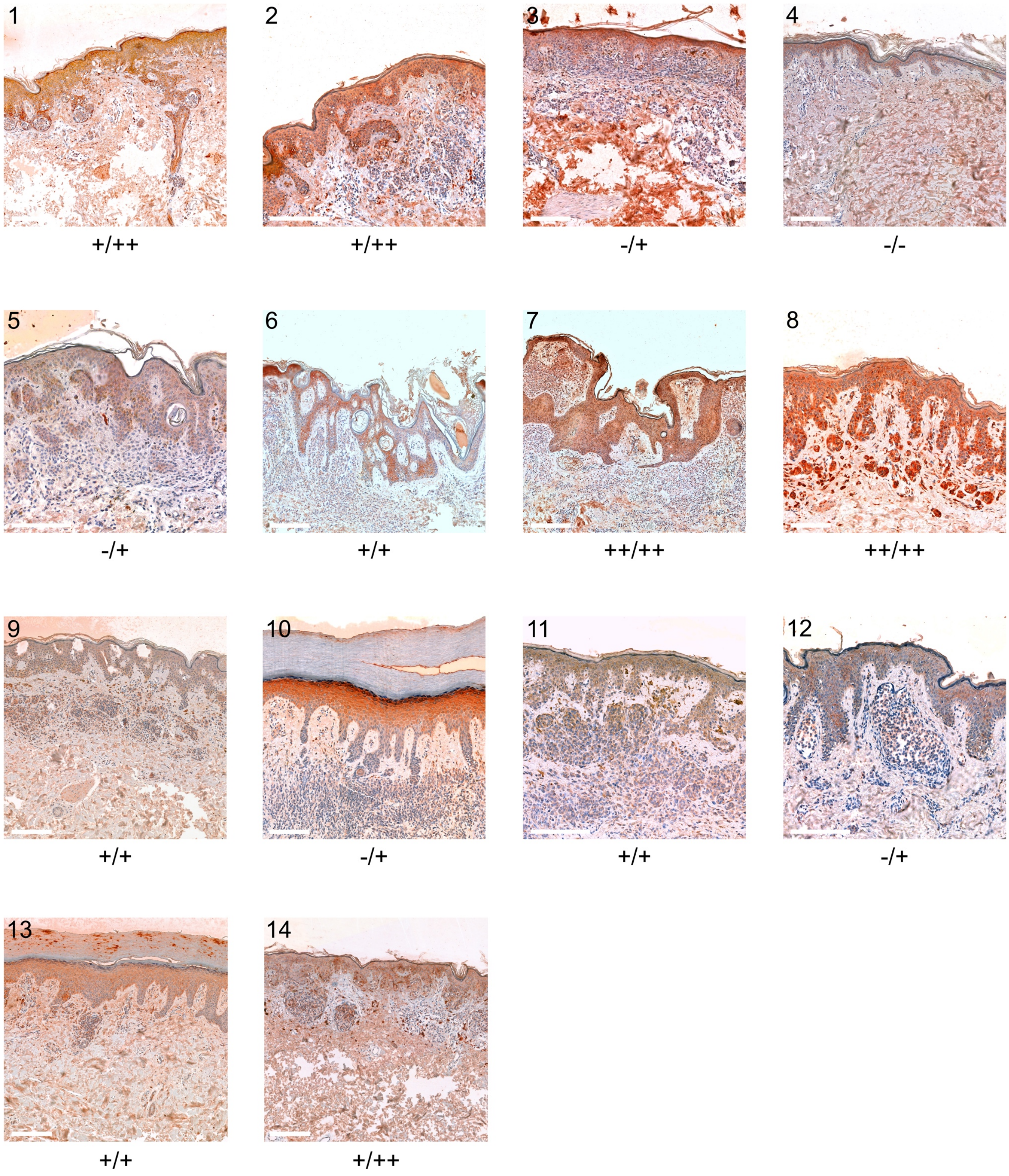

Supplementary Figures S6-S7: Immunohistochemistry for TRPC5 in MM. Scale bars represents 200 μm. In epidermal parts, 6/27 are strong positive (2,5,17,18,19,27) and 13/27 are weak positive (1,6,7,8,9,10,11,14,16,21,22,24,25) and no reactions are displayed on 8/27 (3,4,12,13,15,20,23,26). The dermal sections show strong positive reactions in three samples (2,17,19), weak positive reactions on 13/27 (1,5,6,7,9,10,14,16,18,21,22,25,27) and a negative staining in 11/27 (3,4,8,11,12,13,15,20,23,24,26).

1=MM_2017_11, 2=MM_2668_11, 3=MM_7971_10, 4=MM_14046_10, 5=MM_15640_12, 6=MM_21460_10, 7=MM_23158_06, 8=MM_33661_12, 9=MM_253_12, 10=MM_984_12, 11=MM_24419_06, 12=MM_2125_11, 13=MM_6292_12, 14=MM_8927_09, 15=MM_21438_12, 16=MM_25216_12, 17=1817_11, 18=MM_1944_11, 19=MM_2989_11, 20=MM_7060_09, 21=MM_8190_09, 22=MM_8422_09, 23=MM_4477_09, 24=MM_13145_06, 25=MM_14560_12, 26=MM_15841_10, 27=MM_33253_12

Supplementary Figure S6: Immunohistochemistry for TRPC5 in MM (part 1)

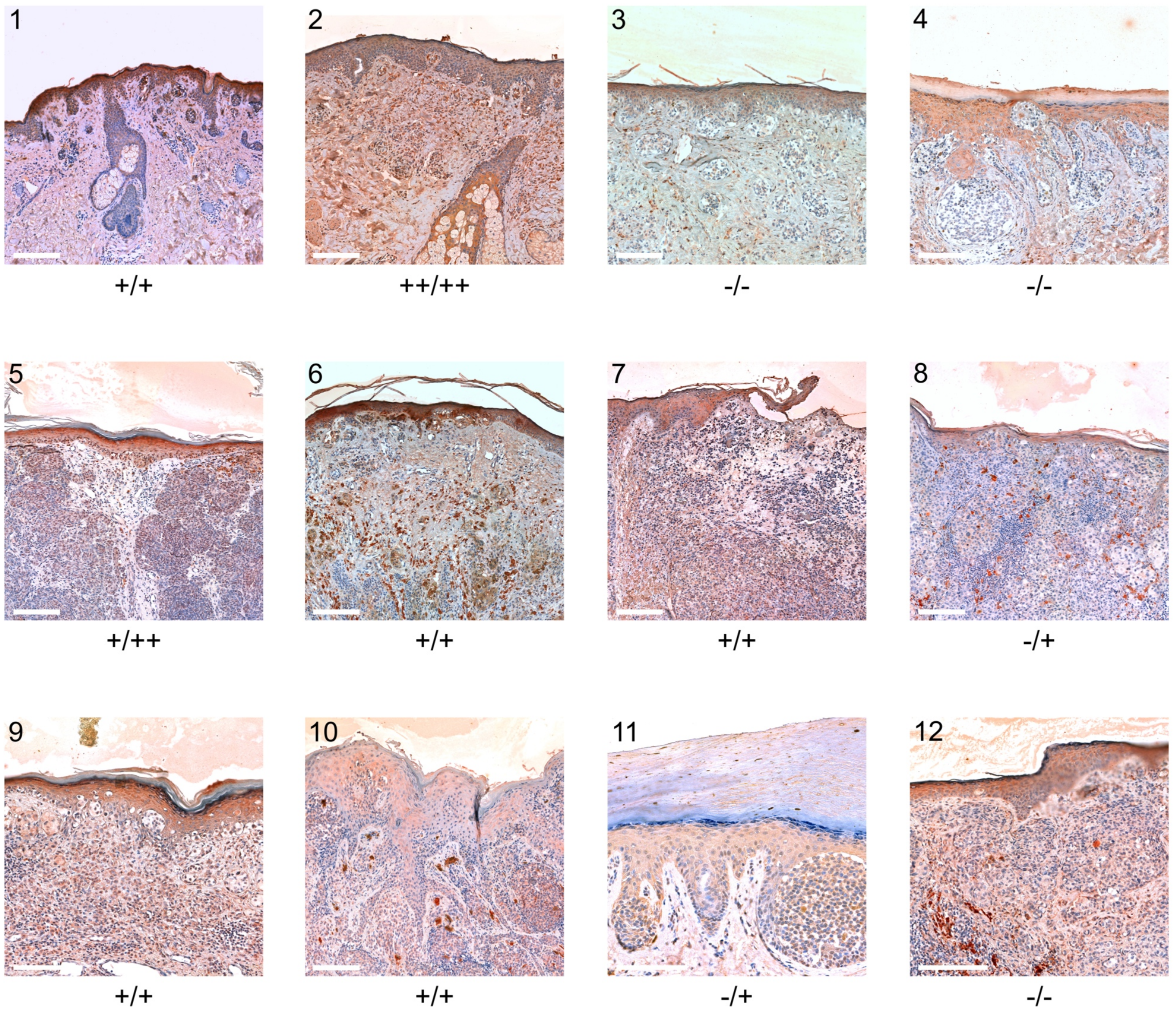

Supplementary Figure S7: Immunohistochemistry for TRPC5 in MM (part 2)

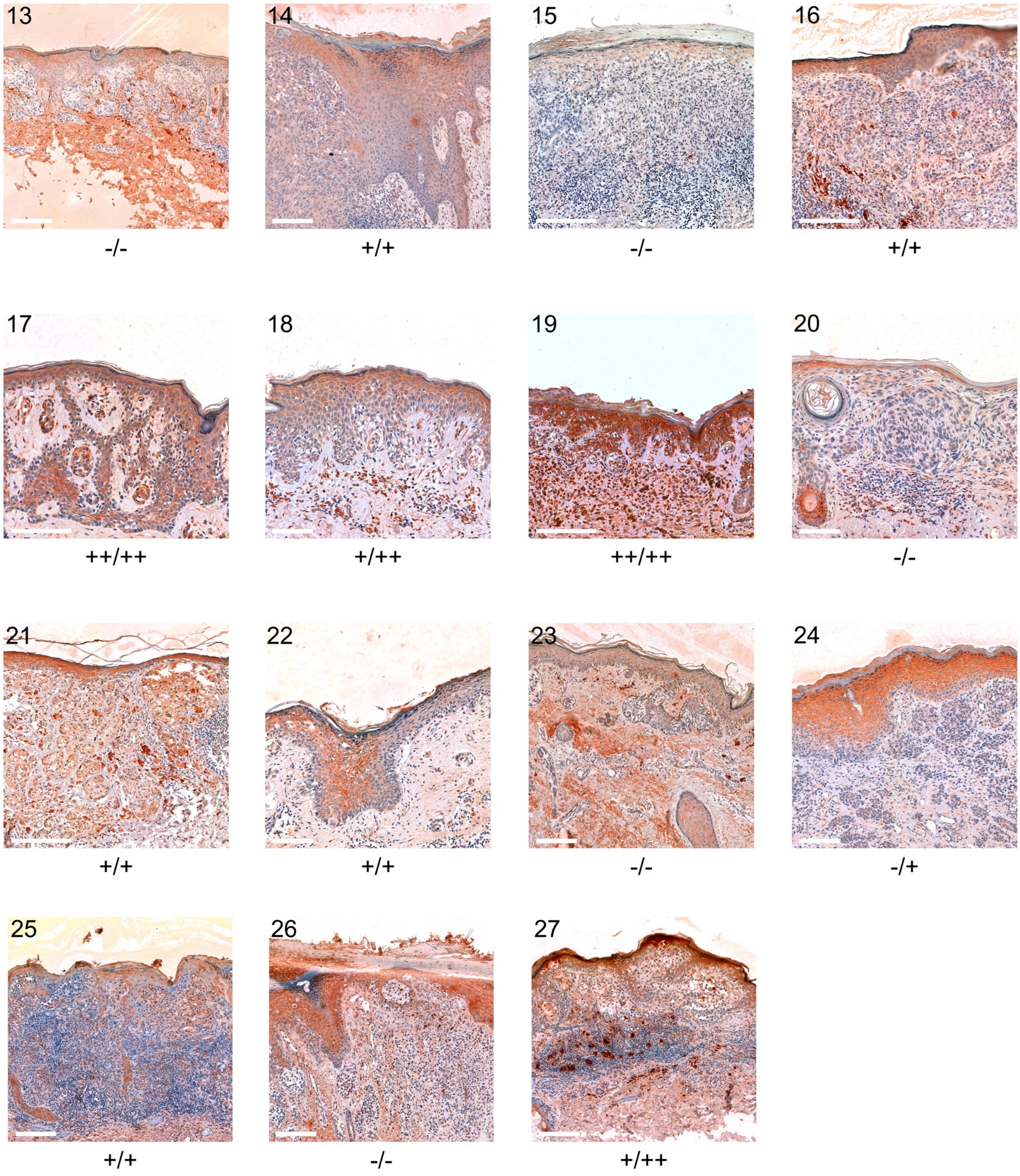

Supplementary Figure S8: TRPC5 mutation frequency in MM from cBioportal.org

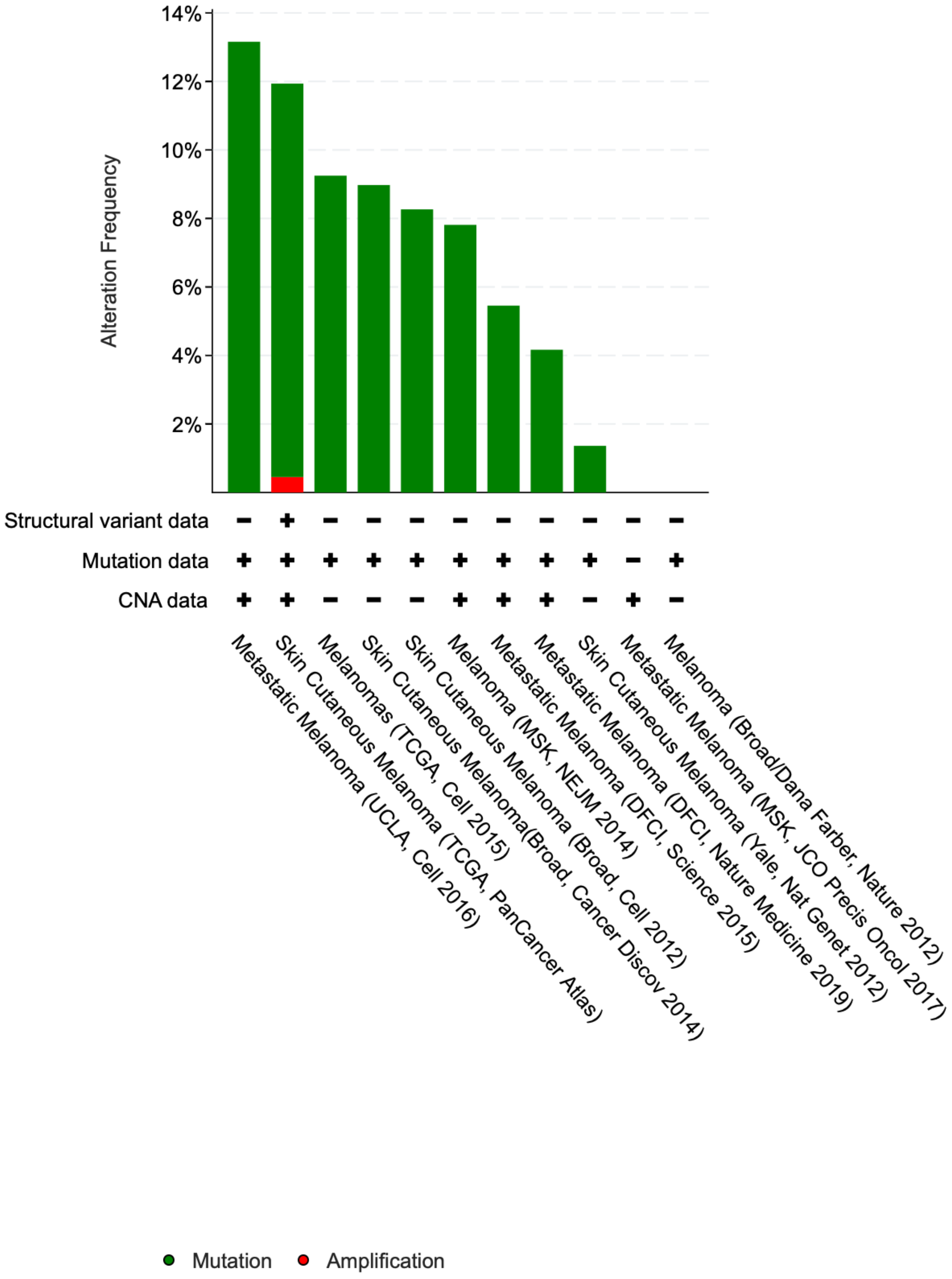

Supplementary Figure S9: TRPC5 Mutation frequency in non-melanoma skin cancer from cBioportal.org

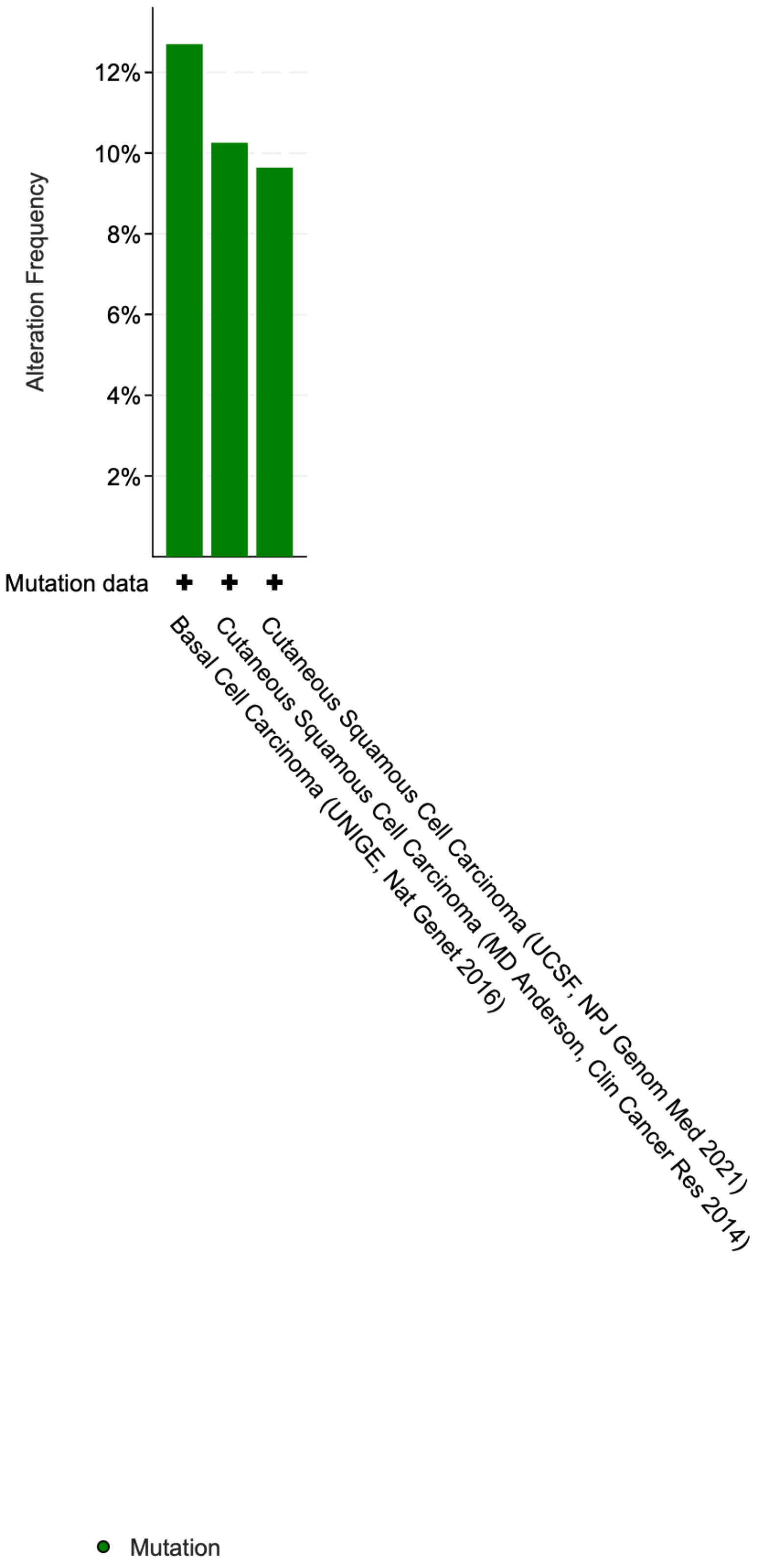

Supplementary Table S1: Scoring results for TRPC5 in BCC

Number	Histo Number	Type of BCC	Scoring
1	BCC_886_12	nodular/sclerosing (mixed)	+
2	BCC_5512_12	nodular	+
3	BCC_8705_12	noduar	+
4	BCC_9027_12	nodular	+
5	BCC_12206_12	nodular	-
6	BCC_12287_12	nodular	+
7	BCC_14077_12	nodular	+
8	BCC_223_09	nodular	-
9	BCC_396_09	nodular	-
10	BCC_1442_06	nodular	-
11	BCC_1786_12	nodular	+
12	BCC_5627_12	superficial	-
13	BCC_845_09	nodular	-
14	BCC_925_09	nodular	+
15	BCC_1121_09	nodular	-
16	BCC_1136_09	nodular	-
17	BCC_2039_09	nodular	-
18	BCC_2388_12	nodular	-
19	BCC_2439_12	nodular	-
20	BCC_252_06	nodular	+
21	BCC_1408_12	nodular	+
22	BCC_8880_12	nodular	+
23	BCC_9013_12	nodular	-
24	BCC_9463_12	nodular	+
25	BCC_9672_12	nodular	+
26	BCC_12207_12	nodular	-
27	BCC_12273_12	nodular	+

Supplementary Table S2: Scoring results for TRPC5 in SCC

Number	Histo Number	Scoring
1	SCC_363_12	++
2	SCC_664_12	-
3	SCC_902_12	+
4	SCC_2090_12	++
5	SCC_9314_09	++
6	SCC_18330_12	++
7	SCC_21605_12	+
8	SCC_24081_12	+
9	SCC_26097_12	++
10	SCC_33497_12	+
11	SCC_2908_09	++
12	SCC_3385_06	++
13	SCC_5682_08	+
14	SCC_11284_06	++
15	SCC_11486_06	+
16	SCC_11824_06	+
17	SCC_13207_06	+
18	SCC_29707_12	-
19	SCC_18011_06	++
20	SCC_16467_06	++
21	SCC_13529_06	++
22	SCC_13160_12	++
23	SCC_11190_09	++
24	SCC_11160_09	++
25	SCC_3385_06	+
26	SCC_1984_12	+

Supplementary Table S3: Scoring results for TRPC5 in NCN

Number	Histo Number	Type of NCN	Scoring dermal	Scoring epidermal
1	NCN_412_12	compound	+	++
2	NCN_11394_12	compound	+	++
3	NCN_11491_12	compound	-	+
4	NCN_11602_12	compound	-	-
5	NCN_11704_12	compound	-	+
6	NCN_17735_09	compound	+	+
7	NCN_26174_12	compound	++	++
8	NCN_37_08	compound	++	++
9	NCN_38_08	compound	+	+
10	NCN_1471_06	acral	-	+
11	NCN_40_08	compound	+	+
12	NCN_290_11	compound	-	+
13	NCN_32_08	compound	+	+
14	NCN_192_12	compound	+	++

Supplementary Table S4: Scoring results for TRPC5 in MM

Number	Histo Number	Scoring dermal	Scoring epidermal
1	MM_2017_11	+	+
2	MM_2668_11	++	++
3	MM_7971_10	-	-
4	MM_14046_10	-	-
5	MM_15640_12	+	++
6	MM_21460_10	+	+
7	MM_23158_06	+	+
8	MM_33661_12	-	+
9	MM_253_12	+	+
10	MM_984_12	+	+
11	MM_24419_06	-	+
12	MM_2125_11	-	-
13	MM_6292_12	-	-
14	MM_8927_09	+	+
15	MM_21438_12	-	-
16	MM_25216_12	+	+
17	MM_1817_11	++	++
18	MM_1944_11	+	++
19	MM_2989_11	++	++
20	MM_7060_09	-	-
21	MM_8190_09	+	+
22	MM_8422_09	+	+
23	MM_4477_09	-	-
24	MM_13145_06	-	+
25	MM_14560_12	+	+
26	MM_15841_10	-	-
27	MM_33253_12	+	++

Supplementary Table S5: Statistical analysis of TRPC5 – comparison of all entities

		Scoring			total
		-	+	++	
SCC	number	2	10	14	26
	% of tumors	7.7%	38.5%	53.8%	100.0%
BCC	number	13	14	0	27
	% of tumors	48.1%	51.9%	0.0%	100.0%
NCN epidermal	number	1	8	5	14
	% of tumors	7.1%	57.1%	35.7%	100.0%
NCN dermal	number	5	7	2	14
	% of tumors	35.7%	50.0%	14.3%	100.0%
MM epidermal	number	8	13	6	27
	% of tumors	29.6%	48.1%	22.2%	100.0%
MM dermal	number	11	13	3	27
	% of tumors	40.7%	48.1%	11.1%	100.0%
Total	number	40	65	30	135
	% of tumors	29.6%	48.1%	22.2%	100.0%

Overall p-value: <0.001

Supplementary Table S6: Patient Data: age and sex of patients the tissue samples stem from

Patient number	Age	Sex
NCN		
1	59	f
2	23	f
3	54	f
4	54	f
5	40	f
6	21	m
7	42	f
8	41	m
9	41	m
10	44	f
11	41	m
12	7	f
13	21	m
14	46	m
MM		
1	46	m
2	33	f
3	30	f
4	83	f
5	83	f
6	42	f
7	59	m
8	77	m
9	55	f
10	66	f
11	24	f
12	69	m
13	43	f
14	82	f
15	48	f
16	47	f
17	56	m
18	51	f
19	71	f
20	69	f
21	56	m
22	63	m
23	59	f
24	75	f
25	62	f
26	111	f
27	43	f
SCC		
1	82	f
2	83	m
3	63	m
4	74	f

5	72	m
6	79	m
7	73	m
8	75	f
9	87	f
10	73	m
11	74	m
12	79	m
13	46	f
14	65	m
15	82	f
16	71	f
17	78	m
18	93	f
18	83	f
20	51	m
21	93	f
22	80	m
23	60	m
24	65	m
25	79	m
26	53	m
BCC		
1	68	m
2	56	m
3	59	m
4	85	m
5	75	m
6	41	m
7	85	f
8	79	f
9	74	m
10	84	f
11	51	f
12	73	m
13	85	m
14	82	m
15	63	m
16	74	f
17	57	m
18	65	f
19	68	m
20	44	f
21	67	m
22	50	f
23	53	m
24	52	f
25	78	m
26	55	f
27	38	f
